# Development and application of Chinese medical ontology for diabetes mellitus

**DOI:** 10.1186/s12911-023-02405-y

**Published:** 2024-01-19

**Authors:** Jie Hu, Zixian Huang, Xuewen Ge, Yulin Shen, Yihan Xu, Zirui Zhang, Guangyin Zhou, Junjie Wang, Shan Lu, Yun Yu, Cheng Wan, Xin Zhang, Ruochen Huang, Yun Liu, Gong Cheng

**Affiliations:** 1 Department of Medical Informatics, School of Biomedical Engineering and Informatics, Nanjing Medical University, Nanjing, Jiangsu China; 2 Department of Information, the First Affiliated Hospital, Nanjing Medical University, No.300 Guang Zhou Road, Nanjing, Jiangsu China; 3 State Key Laboratory for Novel Software Technology, Nanjing University, Nanjing, China; 4 Institute of Medical Informatics and Management, Nanjing Medical University, No.300 Guang Zhou Road, Nanjing, Jiangsu China; 5 Outpatient Department of the First Affiliated Hospital of Nanjing Medical University, No.300 Guang Zhou Road, Nanjing, Jiangsu China

**Keywords:** Diabetes mellitus, Chinese medical ontology, Ontology construction, Question answering, Clinical decision support

## Abstract

**Objective:**

To develop a Chinese Diabetes Mellitus Ontology (CDMO) and explore methods for constructing high-quality Chinese biomedical ontologies.

**Materials and methods:**

We used various data sources, including Chinese clinical practice guidelines, expert consensus, literature, and hospital information system database schema, to build the CDMO. We combined top-down and bottom-up strategies and integrated text mining and cross-lingual ontology mapping. The ontology was validated by clinical experts and ontology development tools, and its application was validated through clinical decision support and Chinese natural language medical question answering.

**Results:**

The current CDMO consists of 3,752 classes, 182 fine-grained object properties with hierarchical relationships, 108 annotation properties, and over 12,000 mappings to other well-known medical ontologies in English. Based on the CDMO and clinical practice guidelines, we developed 200 rules for diabetes diagnosis, treatment, diet, and medication recommendations using the Semantic Web Rule Language. By injecting ontology knowledge, CDMO enhances the performance of the T5 model on a real-world Chinese medical question answering dataset related to diabetes.

**Conclusion:**

CDMO has fine-grained semantic relationships and extensive annotation information, providing a foundation for medical artificial intelligence applications in Chinese contexts, including the construction of medical knowledge graphs, clinical decision support systems, and automated medical question answering. Furthermore, the development process incorporated natural language processing and cross-lingual ontology mapping to improve the quality of the ontology and improved development efficiency. This workflow offers a methodological reference for the efficient development of other high-quality Chinese as well as non-English medical ontologies.

## Introduction

Ontology is a formal and explicit description of concepts and their relationships and has been widely used as a general mode of knowledge representation in the fields of biomedical and health informatics [1, 2]. Applications include clinical and medical natural language processing [3, 4], information retrieval, clinical decision support (CDS) [5], knowledge graphs [6], and medical question answering (MedQA) [7–9].

While the majority of widely used biomedical ontologies are described in English, there is a lack of publicly available, high-quality Chinese ontologies in repositories such as the BioPortal [10], the Open Biological and Biomedical Ontology Foundry (OBO) [11], and the Chinese open knowledge graph community OpenKG.CN (http://www.openkg.cn/dataset), which limits the application of ontologies in Chinese medical scenarios.

The direct translation of existing English ontologies for introduction into China presents several issues, including high human resource costs for medical experts, difficulty in controlling standardization and consistency, incomplete coverage of terminology and semantic relationships, and incompatibility with the clinical practice environment in China [12]. In terms of disease prevention, treatment, nursing, diet, exercise, and management, there are differences between China and other countries. Notably, China possesses unique knowledge related to traditional Chinese medicine and herbal remedies. Therefore, it is essential to develop disease ontologies that are suitable for the Chinese clinical environment. However, work in this area is currently lacking and faces several challenges: (1) There is difficulty in obtaining and sharing the original corpus, and there are few publicly available Chinese medical ontologies. (2) The processes and methodologies for constructing Chinese medical ontologies are not yet mature. (3) Chinese medical experts are not well-versed in ontology and ontology development, making it difficult to assemble a large-scale team for manual construction.

The goals of this study are as follows:

1. The development of an open-source Chinese Diabetes Mellitus Ontology (CDMO). This endeavor is underscored by the significant prevalence of diabetes as a chronic ailment in China, with its potential to engender a multitude of grave complications [13].

In the field of diabetes, Diabetes Mellitus Treatment Ontology (DMTO) is a high-quality English ontology [14], which extends Diabetes Diagnosis Ontology (DDO) [15], oriented to decision support for diagnosis and treatment of type 2 diabetes. The construction methodology of DMTO adopts an extraction approach to reuse some existing ontologies, and a top-down approach to construct hierarchical relationships between classes. Cecilia Reyes-Peña et al. built an Ontology Network to capture and manage ontological and non-ontological information about diabetes mellitus (DM) in Mexico for diagnosis process [16]. The DM Ontology Network composed of six modules, include control plan, clinical entity, education level, clinical information administration, geographic location, and person.

(2) The introduction of a robust framework tailored for the semiautomated generation of high-caliber Chinese medical ontologies. This initiative seeks to curtail the necessity for expert intervention. Furthermore, this framework offers a valuable reference point for crafting medical ontologies in diverse non-English linguistic contexts.
(3) Illustrating the efficacy of CDMO by integrating it into Clinical Decision Support (CDS) and natural language Medical Question Answering (MedQA) systems, representing quintessential applications for medical ontologies.

## Materials and methods

### Ontology construction

#### An overview of the construction framework

**Fig. 1:**
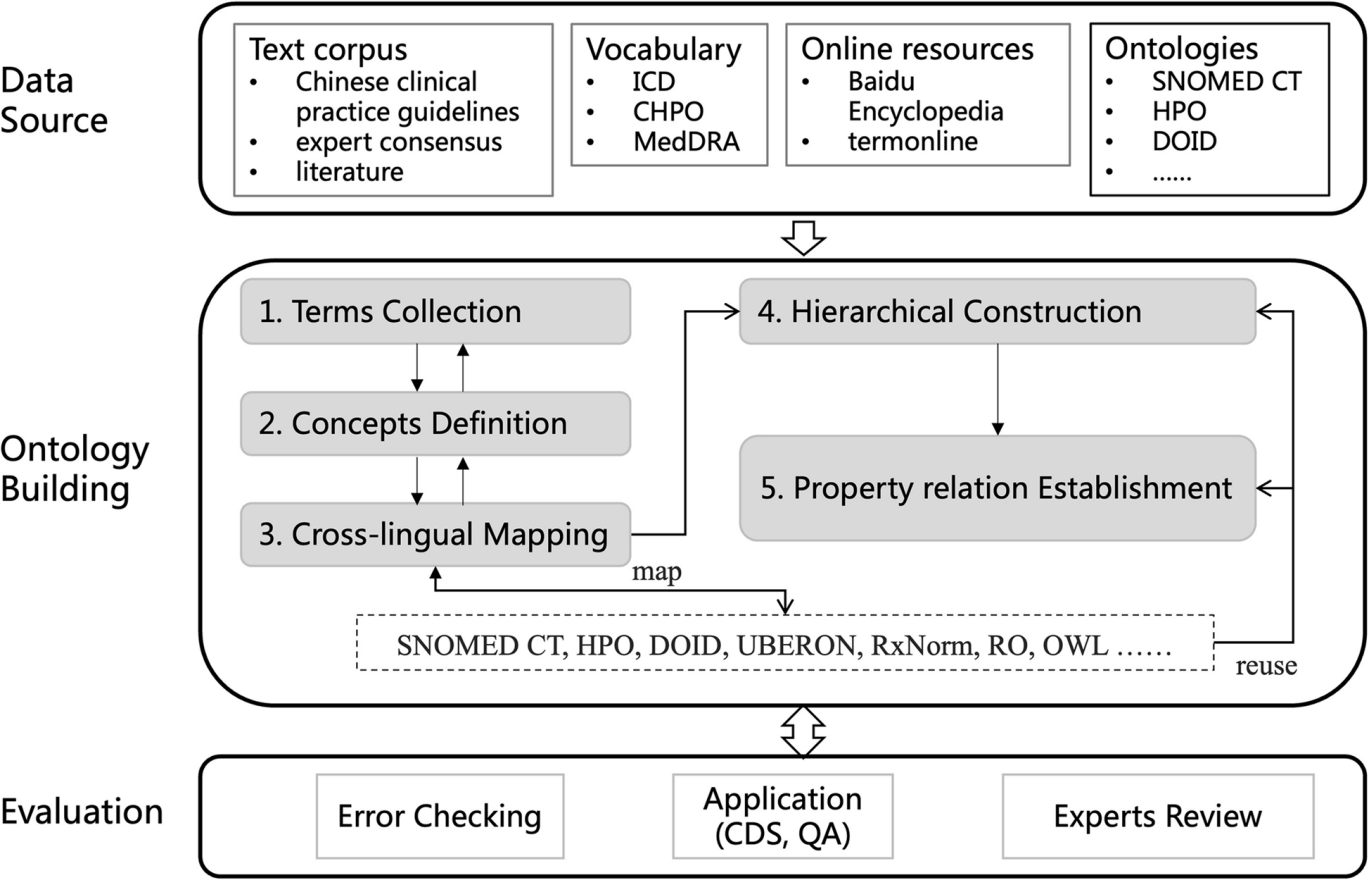
Framework for building Chinese medical ontology

How to construct high-quality Chinese medical ontologies without overburdening medical experts is a challenging problem, and there is still a lack of suitable engineering methodologies that can be widely adopted. We refer to ontology building methods such as UPON [17], UPON Lite [18], and NeON [19], and propose a combination of top-down and bottom-up Chinese medical ontology construction process based on text mining and cross-lingual mapping, which makes it possible to semi-automate the construction of high-quality medical ontologies without excessive reliance on medical experts, the framework is shown in Fig. 1.

### Data source

The data sources utilized in this study comprised (1) Chinese clinical practice guidelines (*n* = 15) and expert consensus (*n* = 68) in the field of diabetes; (2) Chinese diabetes research literature publicly available from the TIANCHI dataset (https://tianchi.aliyun.com/dataset/88836); (3) glossaries of Chinese medical terms, such as International Classification of Diseases (ICD) versions 10 and 11, Medical Dictionary for Regulatory Activities (MedDRA), Chinese Human Phenotype Ontology (CHPO), and dictionaries used in hospital information systems; (4) online resources, including termonline (https://www.termonline.cn) and Baidu Encyclopedia; and (5) widely recognized biomedical ontologies in English for cross-lingual ontology mapping.

### Ontology building

The process of building CDMO is primarily divided into five stages: (1) terms collection, where key Chinese terms in the field of diabetes are obtained through text mining and consultation with domain experts; (2) concept definition, where definition descriptions and synonyms of terms are obtained through online resources or expert consultation; (3) cross-lingual mapping, where these concepts are matched to widely recognized English ontologies; (4) hierarchical construction, where hierarchical relationships between concepts are established based on concept definitions, contextual information from clinical practice guidelines, and mapping results; and (5) property relation establishment, where other non-hierarchical relationships between concepts are established by reusing and identifying new properties of these concepts.

To facilitate the development process, we utilized Webprotégé [20] and Protégé, which are widely recognized as the preeminent environments for ontology development [21].

### Evaluation

CDMO was verified by ROBOT tools [22], expert review, and the application assessment was made through MedQA and CDS.

#### Terms collection

Collecting domain-related terms is a fundamental task in ontology construction. Due to the complexity of Chinese named entity recognition and the difficulty of entity boundary recognition compared to English, we adopted a combination of text mining and manual approaches.

Initially, we used the Aho-Corasick algorithm to identify 4,700 terms from the text corpus based on dictionaries, including MedDRA, CHPO, and Chinese versions of ICD-11 and ICD-10. We excluded terms about grades and degrees, such as “1级” (grade 1), “1期” (stage 1), and “全部” (all), from the terminology set.

Subsequently, we employed a named entity recognition model based on BiLSTM-CRF [23] to extract entities from the text corpus. Due to errors caused by using Optical Character Recognition tools to convert PDF files to plain text and the accuracy of the algorithm itself, this process generated a large amount of noisy data, resulting in a total output of 47,598 entities. After manually removing incorrect terms (e.g., entities with punctuation, simple numerical values, and misspellings) and merging literal synonyms (e.g., “气血足” and “气血两足”, “肾病” and “肾脏病” (kidney disease)), we obtained 6,816 candidate terms.

The results from the above two steps were combined, and obviously irrelevant terms were removed to obtain a total of 9,440 candidate terms. Such irrelevant terms include “窗” (window), “猫” (cat), “家” (home), and “争论” (argument), etc. As in the previous step, some literal synonyms needed to be merged in this step as well.

In step four, to ensure the conciseness of the ontology, we adopted the following strategy to further filter terms that are closely related and important for diabetes: a corresponding entry exists in dictionaries or termonline or in Baidu Encyclopedia, and the term appears in at least three different documents in the corpus. After filtering, a total of 3,342 terms were obtained.

Finally, to improve the domain specificity and coverage of the ontology, we manually extracted terms from three comprehensive clinical guidelines in the field of diabetes, adding a total of 410 terms, such as “随机血糖异常” (random glucose abnormalities) and “A型胰岛素抵抗” (insulin resistance type A), that were not identified in previous steps, as well as genes, units of measurement, foods, drugs, and tests. The three guidelines are Guidelines for the Diagnosis and Treatment of Type 1 Diabetes Mellitus in China (2021 edition), Guideline for the Prevention and Treatment of Type 2 Diabetes Mellitus in China (2020 edition), and National Guidelines for the Prevention and Control of Diabetes in Primary Care (2022 edition).

It is important to differentiate between “substance” and “substance measurement” as they are often used interchangeably in Chinese texts. To address this issue, we have introduced separate terms, such as “肌酐” (creatinine) and “肌酐测定” (creatinine measurement). “肌酐” (creatinine) is a metabolite of creatine found in muscle tissue, while “肌酐测定” (creatinine measurement) is a quantitative assessment of the amount of creatinine present in a sample.

#### Concept definition

Definitions of these terms were imported from authoritative sources such as termonline, CHPO, MedDRA, and Baidu Encyclopedia. The principle of adopting definitions favors termonline over Baidu Encyclopedia, Baidu Encyclopedia over CHPO and CHPO over MedDRA. In termonline, definitions from multiple sources are often provided for a single concept, requiring consideration of temporal priority and specialty characteristics. For example, the concept of “代谢物” (metabolite) adopted the Biophysical Terminology (2nd edition), 2018. In Baidu Encyclopedia, the definition of a term may not be the first sentence of the text describing the term and may require manual processing.

For concepts without available definitions, especially for some clinical tests and clinical findings, English definitions are created based on mapping, translated, and reviewed by clinical experts. For example, the definition of “尿钙” (urine calcium) can be found in NCIT through mapping.

When defining terms, it is important to collect information such as the English name and synonyms of the term, which can be used to merge terms. For instance, “心肌梗塞” (myocardial infarction) is the common name for “心肌梗死” (heart attack) and can be merged. Similarly, “黑棘皮症” and “黑棘皮病” share the same English name, “acanthosis nigricans”, and can be merged.

#### Cross-lingual ontology map

Mapping Chinese terms to widely recognized English ontologies provides three benefits for Chinese ontologies: 1) enhancing interoperability, 2) improving quality, and 3) enabling knowledge sharing. Despite the availability of cross-language ontology mapping tools such as AML [24], this task remains challenging due to the low accuracy of matching results and the need for human verification. For example, in AML, the similarity score between “慢性肾脏病2期” (stage 2 chronic kidney disease) of CDMO and “stage 4 chronic kidney disease” (HP_0012626) of HPO is 0.9338, obviously, this mapping is wrong. Similarly, the similarity score between “视力” (Vision) and “视力模糊” (Blurred vision) calculated by AML is 0.8836, but these are two distinct concepts. Additionally, if the matching ontology is large, such as SNOMED CT, the process is inefficient and requires the purchase of the Azure API.

**Fig. 2:**
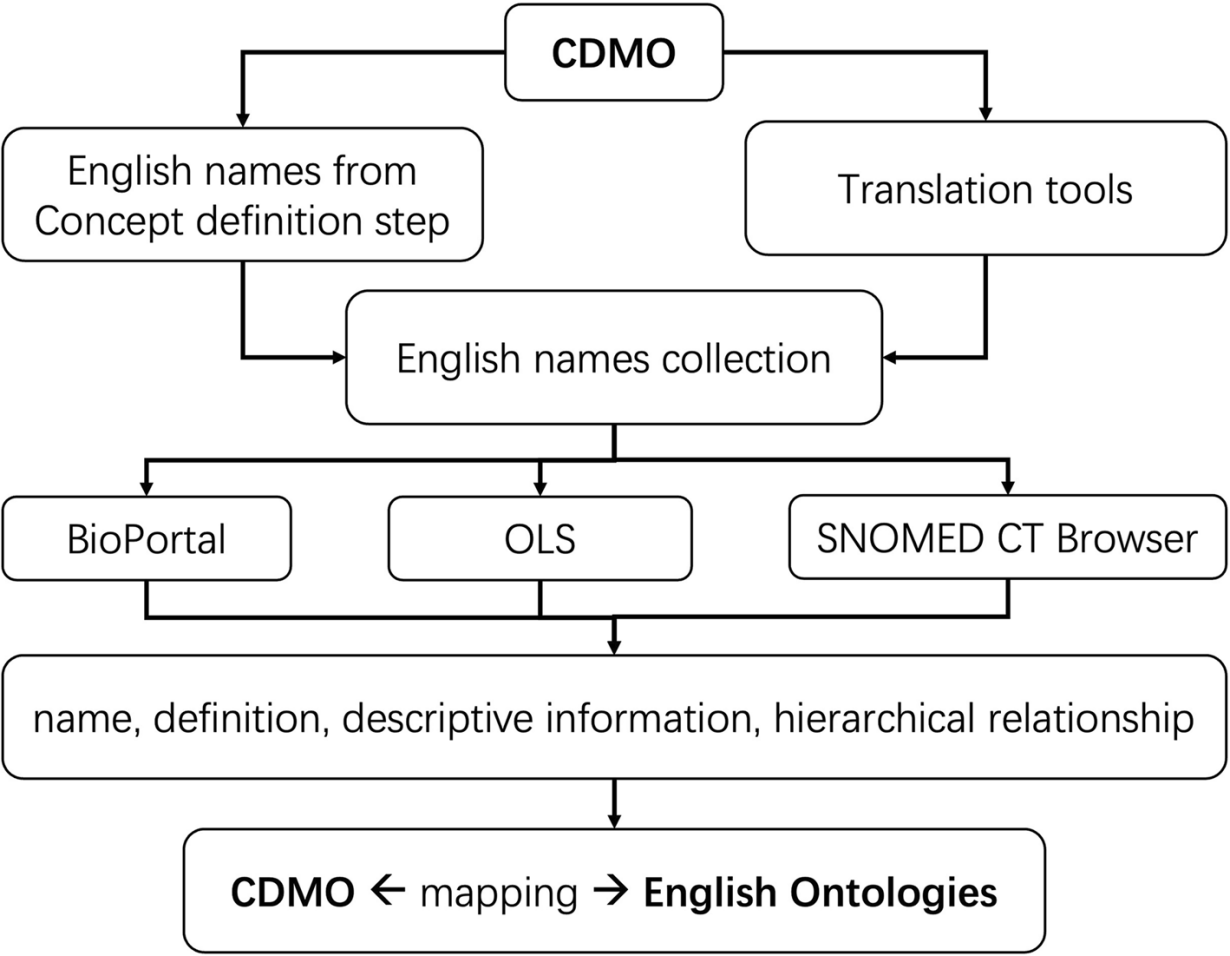
Cross-lingual ontology mapping procedure

To establish CDMO mapping to as many English ontologies as possible, manual mapping was performed with the assistance of translation tools and the Ontology Lookup Service (OLS, https://www.ebi.ac.uk/ols4/). First, a collection of English names for Chinese terms was gathered from concept definition step and translation tools; second, we used OLS, SNOMED CT Browser, and BioPortal to search for suitable mappings based on name, definition, descriptive information, or hierarchical relationships between concepts. The process of cross-lingual mapping is illustrated in Fig. 2.

For instance, the Chinese term “高渗性高血糖状态” has been translated into English as “hyperosmolar hyperglycemic state” by termonline and “hyperglycemic hyperosmolar status” by clinical practice guidelines. Although the two translations differ slightly from each other, neither of them can be found as an exact match in SNOMED CT. However, the corresponding class can be located in BioPortal (MedDRA:10076423). To determine the mapping of this Chinese term to SNOMED CT, we performed a search for "hyperosmolar hyperglycemic" in the SNOMED CT Browser and found the possible mapping object "HHS—Hyperosmolar hyperglycemic syndrome". According to the definition of "高渗高血糖综合征" (Hyperosmolar Hyperglycemic Syndrome) in the Chinese Guidelines for the Diagnosis and Treatment of Type 1 Diabetes Mellitus (Version 2021), we established the mapping of "高渗性高血糖状态" in CDMO to the corresponding concepts in SNOMED CT and MedDRA.

Here's a more general example where the mapping process involves the use of translation tools, concept definitions and the hierarchical structure of the ontology. For the Chinese term “乏力”, termonline provides the English names {malaise, fatigue, weakness, lack of power, acratia}, while Youdao translates it as {lacking in strength, weak, feeble}, Google as {fatigue}, and DeepL as {lack of power, weakness, fatigue}. Another similar term, “疲劳”, is translated as {fatigue} by termonline, {tired, fatigue, weary} by Youdao, {fatigue} by Google, and {fatigue, tiredness, weariness} by DeepL. When these terms are searched in OLS, several different hits are returned. In these complex cases, mapping needed reading both Chinese and English definitions and referring to the hierarchical relationships of the English ontology. For example, in SNOMED CT, terms such as “Asthenia” and “Weakness” are hyponyms of “Fatigue”. As a result, we chose to map the term “疲劳” to “Fatigue” (SCTID: 84229001) in SNOMED CT.

Additionally, distinctions between substances and substance measurements needed to be made during ontology construction and mapping. For example, the term “糖化血红蛋白” (glycated hemoglobin) was mapped as a substance to “glycated hemoglobin-A1c (substance)” (SCTID: 733830002), while “糖化血红蛋白测定” (hemoglobin A1c measurement) was mapped to “hemoglobin A1c measurement (procedure)” (SCTID: 43396009) in SNOMED CT.

The current version of CDMO has 12,360 links to 60 English ontologies. Table 1 shows the specific contents with over 100 mappings. SNOMED CT and NCIT, as comprehensive medical ontologies, have the highest number of mappings.

**Table 1:** External database mappings

Ontology	Map numbers
SNOMED CT	3128
NCIT: NCI Thesaurus OBO Edition	2107
OMIT: Ontology for MIRNA Target	1189
HPO: Human Phenotype Ontology	705
EFO: Experimental Factor Ontology	595
OAE: Ontology of Adverse Events	579
MONDO: Mondo Disease Ontology	555
DOID: Human Disease Ontology	486
CHEBI: Chemical Entities of Biological Interest	373
FMA: Foundational Model of Anatomy Ontology	325
MP: The Mammalian Phenotype Ontology	285
UBERON: Uber-anatomy ontology	270
SYMP: Symptom Ontology	183
GSSO: the Gender, Sex, and Sexual Orientation ontology	132
ORDO: Orphanet Rare Disease Ontology	118
MAXO: Medical Action Ontology	116

#### Hierarchical relationship construction

The hierarchical relationship between concepts is the backbone of an ontology, but constructing it using manual or natural language processing technologies can be challenging. Some hierarchical relationships may not be well understood by clinicians, and even advanced natural language processing techniques can yield incorrect results [25]. In a resource-poor environment such as the Chinese Medical Ontology, deep learning-based approaches are hindered by the lack of credible training data.

To address this challenge, we first adopted the first-level of SNOMED CT as our top-level structure and then employed two methods to construct hierarchical relationships.

**Fig. 3:**
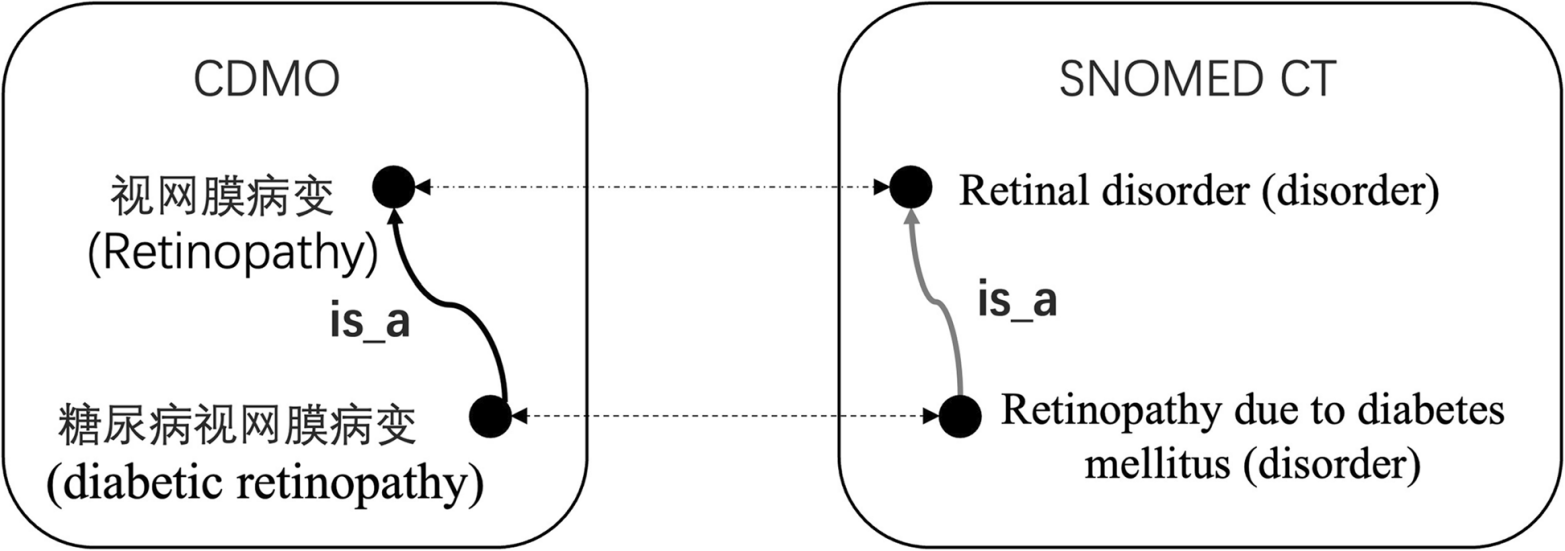
Concept hierarchy construction based on cross-linguistic mapping

The first method involved automatic construction of hierarchical relationships using cross-lingual mapping. Ontologies such as SNOMED CT and NCIT have established relatively good hierarchical relationships that can be exploited through cross-lingual mapping. For instance, as depicted in Fig. 3, the terms “视网膜病变 (retinopathy)” and “糖尿病视网膜病变 (diabetic retinopathy)” of CDMO are mapped to “retinal disorder (disorder)” and “retinopathy due to diabetes (disorder)” of SNOMED CT respectively. Additionally, within SNOMED CT, “retinaopathy due to diabetes (disorder)” is classified as a subtype of “retinal disorder (disorder)” through an *is_a* hierarchical relationship. Therefore, this hierarchical relationship is incorporated into CDMO.

The second method involved manual construction of hierarchical relationships based on the context and definition of concepts. We extracted hierarchical relationships primarily from the context of concepts in authoritative clinical practice guidelines and definitions. For example, one guideline states: “Diabetes mellitus is classified into four types based on etiological evidence, namely type 1 diabetes mellitus (T1DM), type 2 diabetes mellitus (T2DM), special types of diabetes mellitus, and gestational diabetes mellitus.” From this statement, it can be inferred that type 1 diabetes, type 2 diabetes, special types of diabetes, and gestational diabetes are all subtypes of diabetes mellitus. Hierarchical relationships between some concepts can also be inferred from their definitions. For instance, “肾上腺皮质激素” (adrenal cortex hormone) is defined as “Steroid hormones (类固醇激素) produced by the adrenal cortex stimulated by adrenocorticotropic hormones secreted by the pituitary gland. They can be divided into mineralocorticoids (盐皮质激素) and glucocorticoids (糖皮质激素) according to their physiological characteristics.” From this definition, it can be extracted that “glucocorticoid” and “mineralocorticoid” *is_a* “adrenocorticoids” and that “adrenal cortex hormone” *is_a* “steroid hormone”. Additionally, the hierarchy between some concepts can also be inferred from their labels, such as “乳酸性酸中毒” (lactic acidosis) *is_a* “酸中毒” (acidosis).

The construction of hierarchical relationships can provide feedback to the previous two steps by adding new terms and concepts.

#### Non-hierarchical relationship construction

Constructing non-hierarchical relationships is more complex due to their diversity and obscurity. We collected relationships mentioned in other Chinese medical ontology related literature and the OMAHA Schema (https://schema.omaha.org.cn), extracted fine-grained relationships from guidelines, and established hierarchical relationships among them.

1. Annotation Properties

CDMO defines 108 annotation properties, which are divided into five categories: 1) mapping relationships with other ontologies, such as SNOMED CT and NCIT; 2) properties provided in the Simple Knowledge Organization System (SKOS), Resource Description Framework Schema (RDFS), and Web Ontology Language (OWL), such as rdfs:label and skos:definition; 3) properties created for concept annotation, such as “来源语句” (source sentences) and “英文名称” (English names); 4) drug-related annotation information, such as “药物常用剂量” (common drug dosage) and “用药频次” (drug administration frequency); and 5) properties related to test and examination items, such as “正常参考值” (normal reference value) and “诊断切点” (diagnostic cut-off value).

(2) Object Properties

CDMO defines 182 fine-grained object properties and establishes a hierarchy among them. Table 2 provides examples of three groups of object properties: the “并发症” (complications), “治疗药物” (therapeutic drugs), and “症状” (has symptom) properties all have multiple sub-properties.

**Table 2:** A sample of the object properties

Property name	Domain	Range
并发症(has complication) • 常见并发症(has common complications) • 特有的并发症(has specific complications) •急性并发症(has acute complications) • 慢性并发症(has chronic complications) • 近期并发症(has short term complications) • 远期并发症(has long term complications)	临床表现(clinical finding) or 过程(procedure)	临床表现(clinical finding)
治疗药物(therapeutic drug) • 一线药物(first line therapeutic drug) • 二线药物(second line therapeutic drug) • 首选药物(preferred drug) • 慎用药物(cautiously use drug)	临床表现(clinical finding) or 过程(procedure)	药物或生物制品(Pharmaceutical/biologic product) or 药物(drug)
症状(has symptom) • 典型症状(has major feature) • 少见症状(has rare symptom) • 首发症状(has first symptom) • 常见症状(has common symptom)	临床表现(clinical finding)	临床表现(clinical finding)

Finally, 81 object properties were mapped to other ontologies, such as OMAHA Schema, SNOMED CT, NCIT, and RO.

### Ontology evaluation

To assess the quality and usability of CDMO, we combined three distinct evaluation approaches: error checking, expert review, and task fit (QA and CDS) [26].

#### Error checking

We followed the OBO community guidelines and improved the ontology using tools of ROBOT and Protege reasoner, which provide coherent and consistent checks and configurable quality control and manually fixed errors and warnings.

#### Expert review

After constructing the ontology, three clinical experts in the field of diabetes independently reviewed it. Based on their reports, some concepts and attributes were merged, and some errors were fixed.

#### Question answering

Fusing ontological knowledge with pre-trained language models (PLMs) has achieved good results on different tasks, such as QA [27], and using knowledge graphs can improve the quality of language generation [28]. CDMO as a formally representing knowledge of diabetes, and it should be possible to provide background knowledge for the model to improve its performance on natural language MedQA. Here, we take the T5 [29] model as an example to verify the effectiveness of CDMO in the Chinese MedQA scenario.

1. QA Dataset

We collected 12,000 pairs of diabetes-related question–answer pairs from the online medical consultation website (www.familydoctor.com.cn) and manually filtered 7,195 pairs for model training and validation by removing non-response and non-explicit questions.

(2) Model

We choose Randeng-T5-784 M-MultiTask-Chinese [30] as the PLM. It is a T5 model pre-trained for the supervised task of Text2Text unified paradigm on Chinese datasets, and it achieved the 3rd place (excluding humans) on the Chinese zero-shot benchmark ZeroClue, ranking first among all models based on T5.

(3) Experiment

For a question ${\text{Q}}$, we use the BM25 [31] algorithm to retrieve the ${\text{n}}$ most relevant definitions ${{\text{D}}}_{1},\dots ,{{\text{D}}}_{{\text{n}}}$ from ontology as evidences, and catenate them to construct the input of T5 as follows:

$${\text{Question}}:{\text{Q}},\mathrm{ Evidences}: {{\text{D}}}_{1},\dots , {{\text{D}}}_{{\text{n}}}$$

T5 is trained using its standard method with the gradient descent algorithm. We randomly divided the dataset into training (80%), test (20%) and dev (20% of training) datasets. Rouge metrics [32], including Rouge-1, Rouge-2 and Rouge-L, were used as the quantitative evaluation metrics for the similarity between the generated answer text and the standard answers given by doctors. To increase the reliability of our experiment, we repeated the experiment three times with different random seeds.

### Clinical decision support

**Fig. 4:**
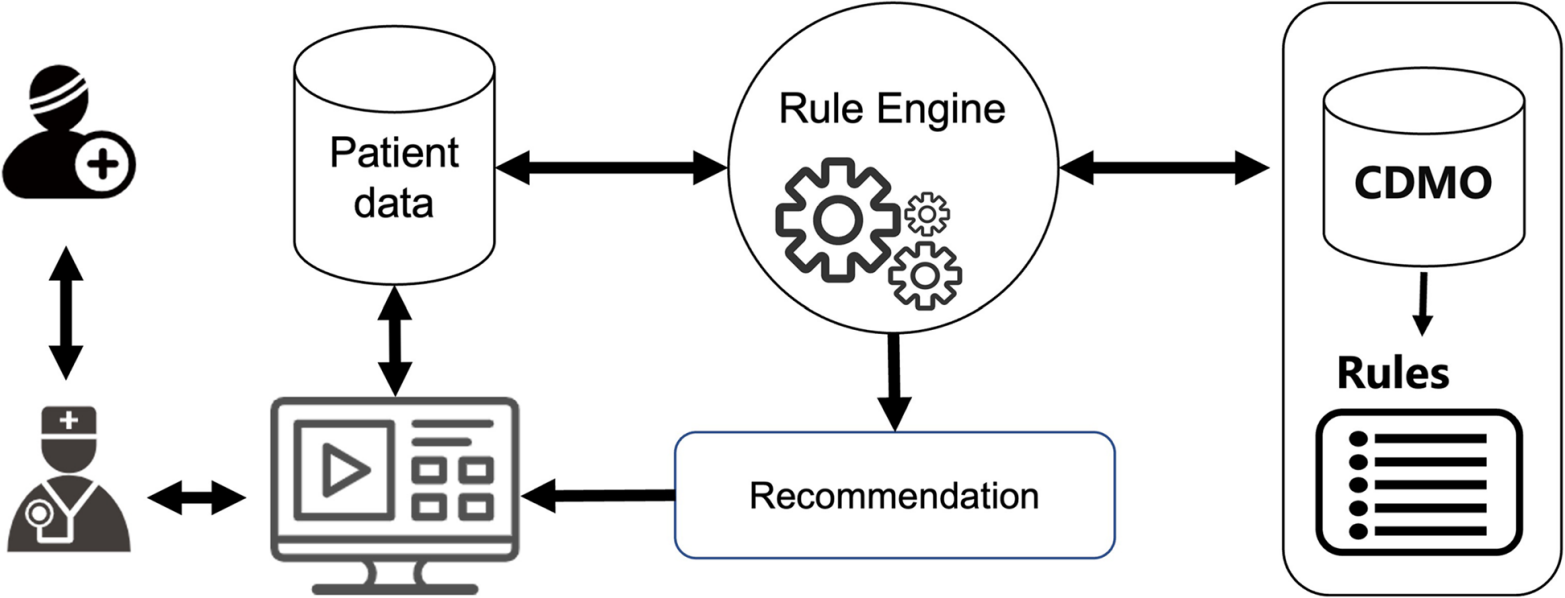
The architecture of the CDS system based on CDMO

CDS is used to provide information and advice to physicians or patients to improve the quality of healthcare. Due to their advantages in knowledge sharing, easy maintenance, reusability, and standardization, ontologies are particularly suitable for clinical guideline modeling and implementation. Based on Chinese diabetes guidelines, CDMO can be used to develop a CDS system for diagnosis and treatment support, as shown in Fig. 4.

The system takes patient symptoms and signs as input, and the inference engine, such as Jena [33], supported by the knowledge representation module, retrieves patient data and performs inference using ontologies and rules. The results of the inference are stored in the database and displayed on the front-end for medical staff to reference.

We developed various rules related to diabetes diagnosis, treatment, diet, exercise and medication recommendations based on clinical practice guidelines for diabetes in China. The rules were developed using CDMO and Semantic Web Rule Language (SWRL) [34] and edited with SWRLTab of Protégé. Table 3 provides examples of these rules. Since CDMO is described in Chinese, this facilitates the conversion of rules described in natural language into SWRL rules, and editors can directly write rules in Chinese. In addition, we keep each natural language rule as a comment message of the rule for review.

**Table 3:** Examples of SWRL rules

Name	Rules represented in SWRL syntax	Explanation of the rule
Diagnosis_1	patient(?p) ^ polyphagia(?s1) ^ polyuria(?s2) ^ polydipsia(?s3) ^ weight_loss(?s4) ^ fasting_blood_glucose_measurement(?t1) ^ diabetes_mellitus(?d1) ^ patient_has_symptom(?p, ?s1) ^ patient_has_symptom(?p, ?s2) ^ patient_has_symptom’(?p, ?s3) ^ patient_has_symptom(?p, ?s4) ^ patient_has_test(?p, ?t1) ^ test_has_value(?t1, ?v1) ^ swrlb:greaterThanOrEqual(?v1, 7.0)—> patient_has_diagnosis(?p, ?d1)	Patients with symptoms of polyphagia, polyuria, polydipsia and weight loss, and fasting blood glucose greater than or equal to 7 mmol/L, are predicted to have diabetes mellitus
Therapy_1	patient(?p) ^ type_2_diabetes_mellitus(?d) ^ body_mass_index(?o) ^ orlistat(?drug1) ^ glucagon_like_peptide_1_receptor_agonist(?drug2) ^ lifestyle_therapy(?therapy) ^ patient_has_diagnosis(?p, ?d) ^ patient_has_observable_entity(?p, ?o) ^ observable_entity_has_value(?o, ?v) ^ swrlb:greaterThanOrEqual(?v, 27)—> patient_has_therapy(?p, ?therapy) ^ patient_has_therapy_drug(?p, ?drug1) ^ patient_has_therapy_drug(?p, ?drug2)	If the patient has type 2 diabetes and the body mass indexis above 27, lifestyle interventions and treatment with glucagon-like peptide 1 receptor agonist and orlistat medications are recommended
Constraindication_drug_1	patient(?p) ^ CDMO:renal_insufficiency(?d1) ^ CDMO:type_2_diabetes_mellitus(?d2) ^ CDMO:metformin(?drug) ^ cdmo:patient_has_diagnosis(?p, ?d1) ^ cdmo:patient_has_diagnosis(?p, ?d2)—> CDMO:patient_has_constraindication_drug(?p, ?drug)	Patients with type 2 diabetes who have renal insufficiency should not take metformin
Diet_1	patient(?p) ^ hypoglycemia(?s1) ^ sugary_food(?f1) ^ glucose(?f2) ^ patient_has_diagnosis(?p,?s1)—> increase_intake(?p, ?f1) ^ increase_intake(?p,?f2)	Patients with hypoglycemia are advised to increase their intake of glucose and sugary foods

## Results

### Ontology metrics

**Fig. 5:**
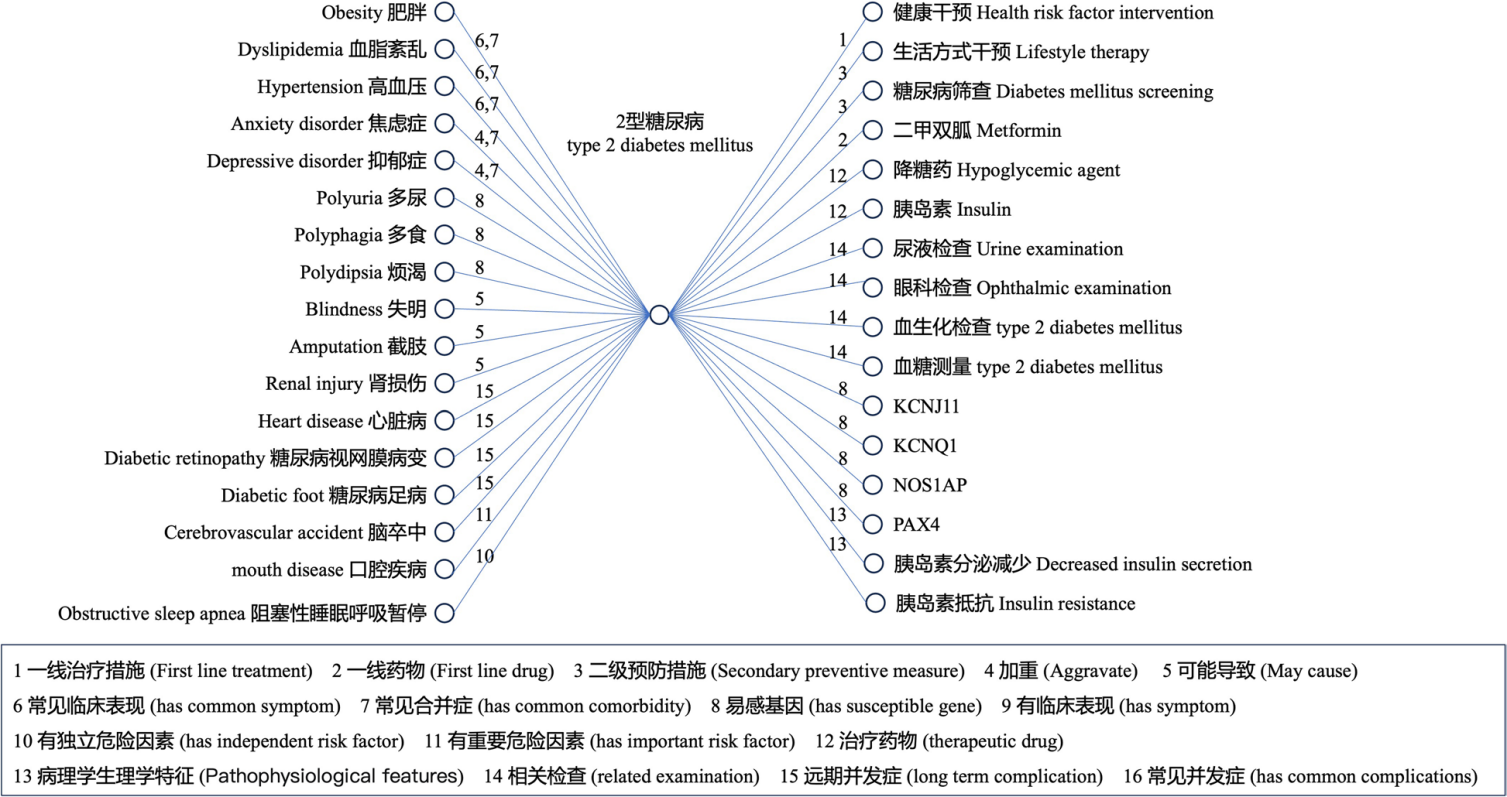
Example of object properties used in CDMO

The current version of CDMO contains 3752 classes, 182 fine-grained object properties with hierarchical relationships, 108 annotation properties with rich information, and 12360 mappings to other well-known English medical ontologies. Figure 5 shows a fragment of the ontology using “type 2 diabetes” as an example, with rich semantic connections through object properties.

1, 一线治疗措施(First line treatment); 2, 一线药物(First line drug); 3, 二级预防措施(Secondary preventive measure); 4, 加重 (Aggravate); 5, 可能导致(May cause); 6, 常见临床表现(has common symptom); 7, 常见合并症(has common comorbidity); 8, 易感基因(has susceptible gene); 9, 有临床表现(has symptom); 10, 有独立危险因素(has independent risk factor); 11, 有重要危险因素(has important risk factor); 12, 治疗药物(therapeutic drug); 13, 病理学生理学特征(Pathophysiological features); 14, 相关检查(related examination); 15, 远期并发症(long term complication); 16, 常见并发症(has common complications).

CDMO has been publicly published on several platforms, including BioPortal, OpenKG and GitHub.

- : https://bioportal.bioontology.org/ontologies
- : https://github.com/HuJieNJ/CDMO
- : http://www.openkg.cn/dataset/cdmo

### Evaluation results

We validated the intrinsic correctness of the ontology using Robot tools and invited three physicians specializing in diabetes to review the ontology's content, including terms, definitions, and relationships.

To verify the value of Chinese medical ontologies in natural language MedQA, we constructed a diabetes domain QA dataset and fine-tuned a T5-based large language pre-trained model by injecting CDMO. The comparison results of injecting ontological knowledge from CDMO are presented in Table 4. On all metrics, CDMO helped the model significantly improve the question answering performance, where CDMO significantly outperformed without CDMO under < 0.01 in both Rouge-2 and Rouge-L, and outperformed without CDMO under < 0.05 in Rouge-1.

**Table 4:** Experimental results of ROUGE scores for the model. We mark the results of with CDMO that are significantly higher than the result of without CDMO under *p* < 0.01 $\left(*\right)$ or *p* < 0.05 $\left(\Delta \right)$

	Dev			Test		
	Rouge-1	Rouge-2	Rouge-L	Rouge-1	Rouge-2	Rouge-L
Without CDMO	32.15	14.38	26.48	32.28	15.00	26.80
With CDMO	$\mathbf{32.81}^{\boldsymbol{\Delta}}$	$\mathbf{14.99}{^\mathbf{\ast}}$	$\mathbf{26.85}{^\mathbf{\ast}}$	$\mathbf{33.13}^{\boldsymbol{\Delta}}$	$\mathbf{15.53}{^\mathbf{\ast}}$	$\mathbf{27.47}{^\mathbf{\ast}}$

**Fig. 6:**
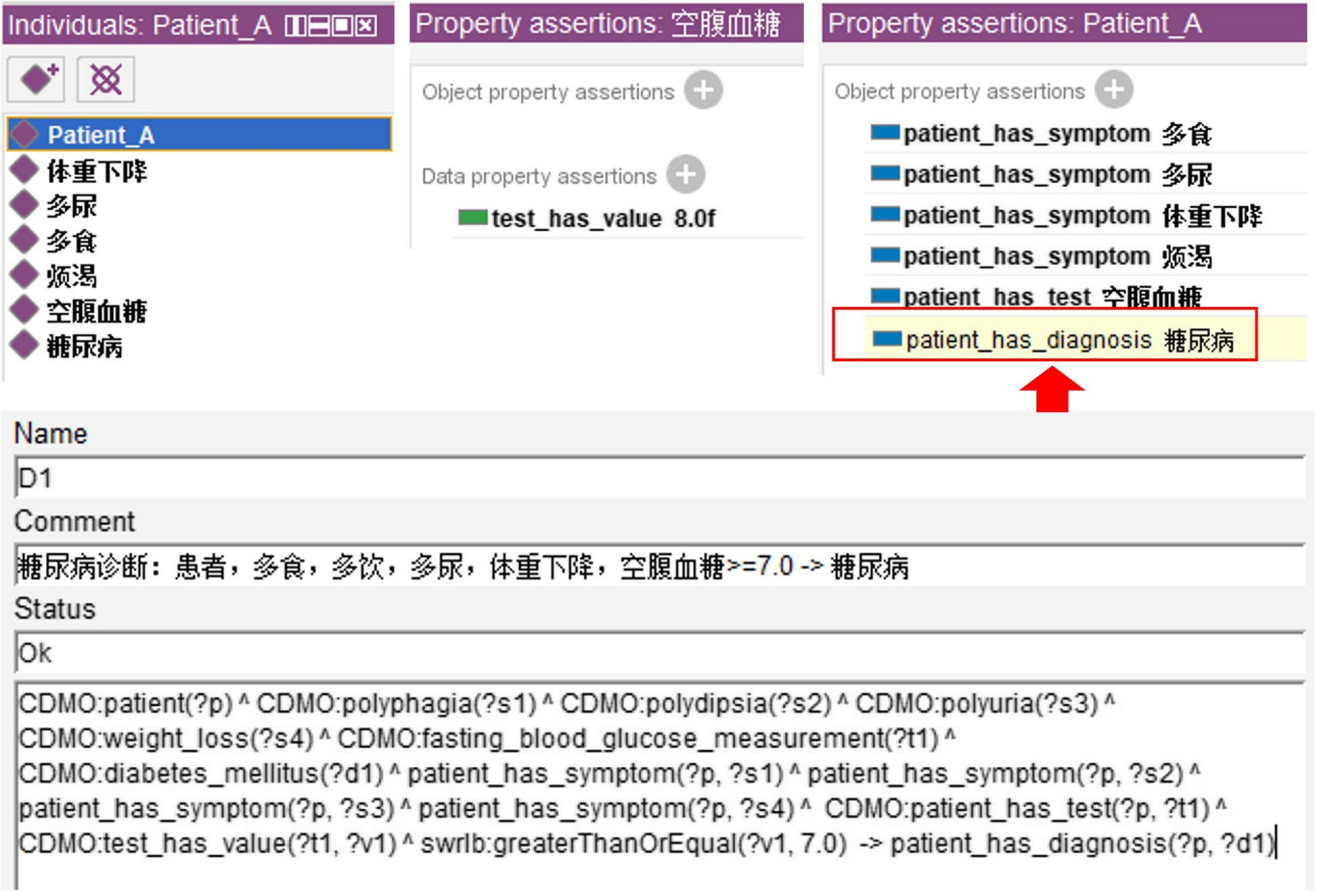
Evaluation of diagnostic rules on simulated instances utilizing the Pellet reasoner

**Fig. 7:**
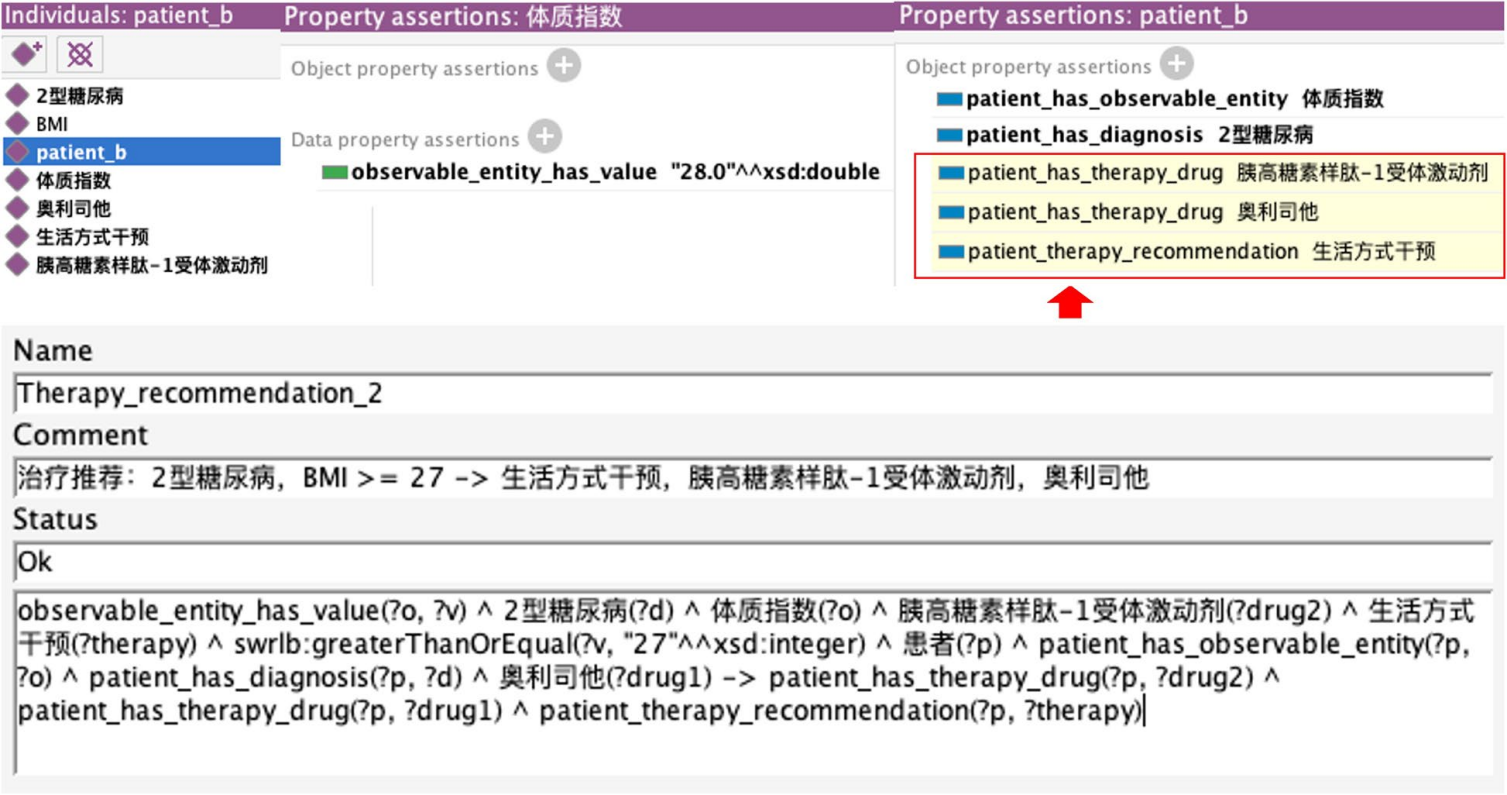
Evaluation of therapy recommendation rules on simulated instances utilizing the Pellet reasoner

We constructed 200 rules related to diabetes diagnosis, treatment, diet, and drug recommendations based on CDMO and clinical guidelines, providing accurate patient case recommendations. We evaluated these rules utilizing Pellet reasoner on simulated ontology instances. The results show that CDMO can be used to express various diabetes decision knowledge, which lays the foundation for the subsequent development of decision support system integrated with hospital information system. As shown in Fig. 6, suppose a patient has the symptoms of weight loss, polyuria, polydipsia and polyphagia, and the test result of fasting blood glucose is 8.0 mmol/L, then the reasoner can infer that he has diabetes mellitus, with the red square indicating. An example of treatment recommendations is shown in Fig. 7. For patient_b with type 2 diabetes and a body mass index (BMI) of 28, it is recommended to consider the use of a glucagon-like peptide-1 receptor agonist (胰高糖素样肽-1受体激动剂) in addition to lifestyle interventions (生活方式干预) based on the SWRL rule.

## Discussion

### Contributions

In this study, we constructed CDMO encoded in OWL by mining and integrating multiple sources of authoritative Chinese diabetes mellitus knowledge, mapped it with numerous English ontologies, and validated it in CDS and MedQA. Our study has three main contributions:

1. We designed a disease-centered Chinese medical ontology construction workflow. Compared with the construction of English medical ontologies, Chinese medical ontologies still lack a number of high-quality ontologies for reuse. We utilizing natural language processing, cross-linguistic cross-ontology matching, and knowledge integration to improve the efficiency and quality of ontology construction while minimizing experts’ workload. Experts only participate in validation and improvement at the end of the construction cycle.
2. We contributed a high-quality medical ontology to the Chinese open-source knowledge base. According to our search, there are no other publicly available Chinese disease ontologies with comparable detail. Compared with the existing English-language ontology in the field of diabetes, the CDMO covers a wider range of diabetes subtypes, complications and diabetes-related diseases, involves diabetes prevention, diagnosis, treatment, patient management and follow-up, and establishes a significant amount of knowledge on Chinese medicine, herbal medicine and dietary aspects with Chinese specialties. Some classes serve as supplements to other English ontologies, such as the class “for'steroid drug use history”.
3. We demonstrated the value of ontologies in professional domain AI application scenarios from two perspectives: CDS and natural language MedQA. The experimental results indicate that CDMO can be used for modelling clinical practice guidelines to build decision support systems, can be used as high-quality knowledge combined with a general language model to enhance the automatic MedQA capability in specific medical domains, and lays the foundation for further construction of a high-quality Chinese-language diabetes knowledge graph.

### Limitations and future effort

During the construction of large-scale medical ontologies, existing algorithms and models for named entity recognition, fine-grained semantic relationship construction, and cross-lingual ontology matching generate numerous errors. This requires significant human effort for screening and selection, making it challenging to improve the automation of ontology construction while ensuring quality.

During the terminology collection phase, we employed the classic deep learning method, BiLSTM-CRF, for Named Entity Recognition (NER) to identify out-of-dictionary terms. A total of 47,598 entities were recognized. However, after manual screening, only 6,816 entities (14.32%) were incorporated into the list of candidate terms. This indicates that there is significant room for improvement in the performance of generic language models when applied to NER for ontology construction in the medical domain.

During the concept definition phase, we observed variations in the descriptions of the same concept across different sources. While we established rules to select a single definition for each concept, such linguistic diversity and synonymy serve as vital data sources for understanding medical language and embedding representations of medical concepts. Future work can delve deeper into exploiting these sources.

For cross-language ontology matching, we applied AML to carry out some experiments, and the results of manual validation were not satisfactory and not very helpful for saving manpower. There are many concepts in CDMOs that have not yet been mapped to the English ontology, including differences in medical knowledge between China and the West, and the fact that English ontologies are in a constant state of renewal. Future directions for continued research may include 2 kinds, one is to develop a user-friendly cross-language mapping software platform to improve efficiency with the help of English names given by medical dictionaries, multiple translation tools and platforms such as OLS; the other is to learn Chinese-English ontology representation with the help of the burgeoning large language model, but this may require a large amount of open-source, high-quality training corpus, and the more than 10,000 mappings that have already been established in this study can provide assistance for this purpose.

Cross-language ontology-based matching can reuse hierarchical relationships between concepts in English ontologies but faces 2 challenges: 1) the quality of matching, and 2) how to address the inconsistency of hierarchical relationships between English ontologies.

To increase practical use, ontologies must be combined with other knowledge representation methods, such as ontology-based rules. Since CDMO is expressed in Chinese, this facilitates ontology-based rule set development and evaluation. However, the current construction of clinical rules is primarily manual. Integrating the CDS system with hospital's Electronic Medical Record or Hospital Information System would allow for large-scale verification in practical applications and further quality improvement through user feedback. While the ontology constructed in this study was not specifically designed for natural language question answering, using it to enhance the performance of MedQA warrants further research.

A good ontology requires maintenance and updates over time. We will continue to optimize and expand CDMO by leveraging developments in areas such as ontology building and large pre-trained language models.

## Conclusion

In this paper, we introduce CDMO, which features fine-grained object properties, extensive knowledge source information, and cross-lingual ontology mapping. We extracted a workflow for constructing Chinese disease ontologies that can be generalized to other diseases. The CDMO can be applied in areas such as CDS, MedQA, and knowledge graph construction related to diabetes. Our future goal is to explore better ways to integrate ontologies with large language models to build a more accurate and practical Chinese diabetes QA system. We also aim to develop a CDS system with practical value based on CDMO and a knowledge graph with richer entity information.

## Data Availability

The ontology constructed in the study is publicly available at https://bioportal.bioontology.org/ontologies/CDO, https://github.com/HuJieNJ/CDMO, and http://www.openkg.cn/dataset/cdmo
